# A comparative study of metformin and nicotinamide riboside in alleviating tissue aging in rats

**DOI:** 10.1093/lifemedi/lnac045

**Published:** 2022-10-26

**Authors:** Lingling Geng, Bin Zhang, Haisong Liu, Si Wang, Yusheng Cai, Kuan Yang, Zhiran Zou, Xiaoyu Jiang, Zunpeng Liu, Wei Li, Zeming Wu, Xiaoqian Liu, Qun Chu, Guang-Hui Liu, Jing Qu, Weiqi Zhang

**Affiliations:** Advanced Innovation Center for Human Brain Protection, National Clinical Research Center for Geriatric Disorders, Xuanwu Hospital Capital Medical University, Beijing 100053, China; Aging Translational Medicine Center, International Center for Aging and Cancer, Beijing Municipal Geriatric Medical Research Center, Xuanwu Hospital, Capital Medical University, Beijing 100053, China; State Key Laboratory of Membrane Biology, Institute of Zoology, Chinese Academy of Sciences, Beijing 100101, China; Institute for Stem Cell and Regeneration, Chinese Academy of Sciences, Beijing 100101, China; University of Chinese Academy of Sciences, Beijing 100049, China; Beijing Institute for Stem Cell and Regenerative Medicine, Beijing 100101, China; School of Biomedical Sciences, Hunan University, Changsha 410082, China; Advanced Innovation Center for Human Brain Protection, National Clinical Research Center for Geriatric Disorders, Xuanwu Hospital Capital Medical University, Beijing 100053, China; Aging Translational Medicine Center, International Center for Aging and Cancer, Beijing Municipal Geriatric Medical Research Center, Xuanwu Hospital, Capital Medical University, Beijing 100053, China; The Fifth People’s Hospital of Chongqing, Chongqing 400062, China; State Key Laboratory of Membrane Biology, Institute of Zoology, Chinese Academy of Sciences, Beijing 100101, China; Institute for Stem Cell and Regeneration, Chinese Academy of Sciences, Beijing 100101, China; Beijing Institute for Stem Cell and Regenerative Medicine, Beijing 100101, China; CAS Key Laboratory of Genomic and Precision Medicine, Beijing Institute of Genomics, Chinese Academy of Sciences, Beijing 100101, China; China National Center for Bioinformation, Beijing 100101, China; University of Chinese Academy of Sciences, Beijing 100049, China; Sino-Danish College, University of Chinese Academy of Sciences, Beijing 101408, China; Sino-Danish Center for Education and Research, Beijing 101408, China; State Key Laboratory of Membrane Biology, Institute of Zoology, Chinese Academy of Sciences, Beijing 100101, China; Institute for Stem Cell and Regeneration, Chinese Academy of Sciences, Beijing 100101, China; University of Chinese Academy of Sciences, Beijing 100049, China; Beijing Institute for Stem Cell and Regenerative Medicine, Beijing 100101, China; State Key Laboratory of Membrane Biology, Institute of Zoology, Chinese Academy of Sciences, Beijing 100101, China; Institute for Stem Cell and Regeneration, Chinese Academy of Sciences, Beijing 100101, China; University of Chinese Academy of Sciences, Beijing 100049, China; Beijing Institute for Stem Cell and Regenerative Medicine, Beijing 100101, China; State Key Laboratory of Stem Cell and Reproductive Biology, Institute of Zoology, Chinese Academy of Sciences, Beijing 100101, China; Institute for Stem Cell and Regeneration, Chinese Academy of Sciences, Beijing 100101, China; University of Chinese Academy of Sciences, Beijing 100049, China; Beijing Institute for Stem Cell and Regenerative Medicine, Beijing 100101, China; Advanced Innovation Center for Human Brain Protection, National Clinical Research Center for Geriatric Disorders, Xuanwu Hospital Capital Medical University, Beijing 100053, China; Aging Translational Medicine Center, International Center for Aging and Cancer, Beijing Municipal Geriatric Medical Research Center, Xuanwu Hospital, Capital Medical University, Beijing 100053, China; State Key Laboratory of Stem Cell and Reproductive Biology, Institute of Zoology, Chinese Academy of Sciences, Beijing 100101, China; Institute for Stem Cell and Regeneration, Chinese Academy of Sciences, Beijing 100101, China; Beijing Institute for Stem Cell and Regenerative Medicine, Beijing 100101, China; State Key Laboratory of Stem Cell and Reproductive Biology, Institute of Zoology, Chinese Academy of Sciences, Beijing 100101, China; Institute for Stem Cell and Regeneration, Chinese Academy of Sciences, Beijing 100101, China; Beijing Institute for Stem Cell and Regenerative Medicine, Beijing 100101, China; State Key Laboratory of Stem Cell and Reproductive Biology, Institute of Zoology, Chinese Academy of Sciences, Beijing 100101, China; Institute for Stem Cell and Regeneration, Chinese Academy of Sciences, Beijing 100101, China; Beijing Institute for Stem Cell and Regenerative Medicine, Beijing 100101, China; The Fifth People’s Hospital of Chongqing, Chongqing 400062, China; Advanced Innovation Center for Human Brain Protection, National Clinical Research Center for Geriatric Disorders, Xuanwu Hospital Capital Medical University, Beijing 100053, China; State Key Laboratory of Membrane Biology, Institute of Zoology, Chinese Academy of Sciences, Beijing 100101, China; Institute for Stem Cell and Regeneration, Chinese Academy of Sciences, Beijing 100101, China; University of Chinese Academy of Sciences, Beijing 100049, China; Aging Translational Medicine Center, International Center for Aging and Cancer, Beijing Municipal Geriatric Medical Research Center, Xuanwu Hospital, Capital Medical University, Beijing 100053, China; Beijing Institute for Stem Cell and Regenerative Medicine, Beijing 100101, China; State Key Laboratory of Stem Cell and Reproductive Biology, Institute of Zoology, Chinese Academy of Sciences, Beijing 100101, China; Institute for Stem Cell and Regeneration, Chinese Academy of Sciences, Beijing 100101, China; University of Chinese Academy of Sciences, Beijing 100049, China; Beijing Institute for Stem Cell and Regenerative Medicine, Beijing 100101, China; CAS Key Laboratory of Genomic and Precision Medicine, Beijing Institute of Genomics, Chinese Academy of Sciences, Beijing 100101, China; China National Center for Bioinformation, Beijing 100101, China; Institute for Stem Cell and Regeneration, Chinese Academy of Sciences, Beijing 100101, China; University of Chinese Academy of Sciences, Beijing 100049, China; Aging Translational Medicine Center, International Center for Aging and Cancer, Beijing Municipal Geriatric Medical Research Center, Xuanwu Hospital, Capital Medical University, Beijing 100053, China; Sino-Danish College, University of Chinese Academy of Sciences, Beijing 101408, China

**Keywords:** metformin, nicotinamide riboside, aging, senescence, inflammation

## Abstract

Metformin (MET) and nicotinamide riboside (NR) have both been reported to exert geroprotective effects in multiple species. However, the mechanism by which MET and NR regulate the aging program and delay aging in multiple tissues remains unclear. Here, we demonstrated that MET and NR attenuate aging features in human mesenchymal stem cells. Moreover, by systematically investigating the pathophysiological changes in different tissues from aged rats after oral administration of MET and NR, we showed that both MET and NR treatment alleviated various aging-related characteristics in multiple tissues, including inflammation, fibrosis, and protein aggregates. Consistently, MET or NR treatment partially rescued aging-related gene expression changes in aged rats. Collectively, we report that both MET and NR attenuate senescence phenotypes in human stem cells *in vitro* and in a variety of rodent tissues *in vivo*, thus providing a valuable resource and foundation for further evaluation of these two compounds against aging.

## Introduction

Aging is defined as a chronic process accompanied by dysregulation of tissue homeostasis, progressive functional impairment, and increased morbidity [1, 2]. Typically, chronic inflammation, mitochondrial dysfunction, and metabolic dysregulation are strongly associated with aging [3–5]. In addition, reduced numbers and dysfunction of stem cells also contribute to impaired tissue regeneration and homeostasis, resulting in aging defects at the tissue and organismal levels [4]. Aging is also malleable, and some conserved and inherent biological mechanisms can be targeted for therapeutic interventions to regulate aging or delay aging-related characteristics [6–8].

Small molecule chemicals like metformin (MET) and nicotinamide riboside (NR), which play regulatory roles in various metabolic pathways, have been reported to show protective effects against aging-related properties [9–11]. MET, a widely prescribed antidiabetic drug, targets aging-related molecular mechanisms, including, but not limited to, preserving the normal function of mitochondria and alleviating chronic inflammation [9, 12]. In terms of targets, MET functions through multiple molecular signaling pathways, such as activating the adenosine monophosphate-activated protein kinase, positively regulating antioxidant transcription factor NRF2 [10], inhibiting the mammalian target of rapamycin, and suppressing the insulin-like growth factor pathways [13, 14]. Nicotinamide adenine dinucleotide (NAD^+^) is an essential coenzyme that plays an important role in various metabolic pathways like reduction–oxidation reactions. NAD^+^ is also a co-substrate for other enzymes, including the longevity proteins sirtuins and polyadenosine diphosphate-ribose polymerases [15]. Treatment with the NAD^+^ precursor NR enhances mitochondrial and stem cell functions, thus alleviating aging-associated phenotypes [11]. However, despite these progresses, MET- or NR-related studies typically focused on a single or a few tissues, whereas aging is a progressive process strongly associated with the dysfunction of various tissues. Thus, it is essential to address, for a given species like the rat, whether MET and NR have equal geroprotective influences on each tissue or show a preference for target tissues.

Despite their quite different molecular targets, MET and NR have been reported to display similar geroprotective effects in mice under certain circumstances [11, 16], suggesting that these drugs might share a similar inherent gene-regulatory mechanism against aging. Therefore, it would be interesting to apply a comparative analysis of MET- and NR-treated aged tissues from a systemic perspective to unveil their common gene-modulating patterns and geroprotective mechanisms. However, such studies have not been reported, and the impact of MET and NR, especially the commonality and specificity of their effects on aged individuals across different tissues, remains largely elusive.

Here, we showed that MET and NR exhibited a geroprotective effect on aged human stem cells and various tissues in aged rats. Both MET and NR repressed systemic inflammation and inhibited aging markers across multiple tissues in aged rats. MET and NR treatment attenuated aging-associated gene expression changes across tissues, with the most protective effects in adipose and aortic tissues. Our data reveal the common and specific properties of these two geroprotective compounds in mitigating systemic tissue aging, and serve as a resource for exploring the geroprotective mechanisms underlying the effects of MET and NR.

## Results

### MET and NR alleviate human cell senescence *in vitro*

**Figure 1.**
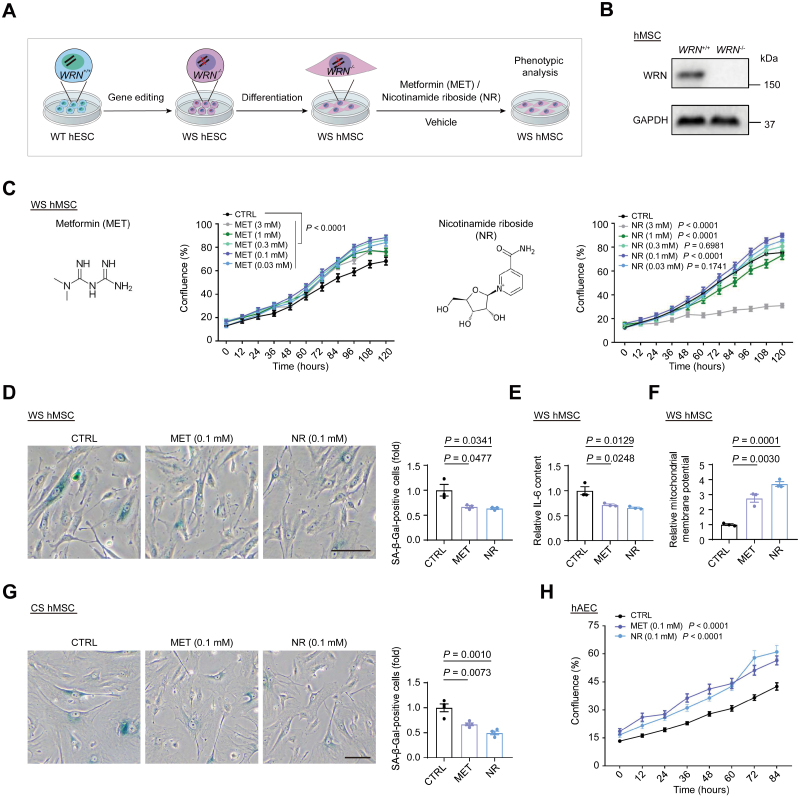
**The geroprotective effects of MET and NR on cell senescence.** (A) Schematic for analysis of the efficacy of MET and NR *in vitro*. (B) Representative Western blot image of WRN protein in *WRN*^+/+^ and *WRN*^−/−^ hMSCs (passage 7). (C) Relative cell proliferation with MET and NR treatment at given concentrations in WS hMSCs (passage 7) using the IncuCyte S3 live cell imaging system (mean ± SD. *n* = 6 biological replicates per group). (D) Representative images (left) and statistics (right) of SA-β-Gal-positive MET-, NR-treated and control WS hMSCs (passage 7) (scale bar, 100 μm; mean ± SEM of ≥ 300 cells from 3 biological replicates; unpaired Student’s *t*-test). (E) ELISA of IL-6 in MET-, NR-treated, and control WS hMSCs (passage 7) (mean ± SEM, *n* = 3 per group; unpaired Student’s *t*-test). Concentrations of both MET and NR are 0.1 mM. (F) Relative MMPs assessed by FACS with JC-10 in MET-, NR-treated, and control WS hMSCs (passage 7) (mean ± SEM. *n* = 3 biological replicates; unpaired Student’s *t*-test). Concentrations of both MET and NR are 0.1 mM. (G) Representative images (left) and statistics of SA-β-Gal staining (right) of MET-, NR-treated, and control CS hMSCs (scale bar, 100 μm; mean ± SEM of ≥ 300 cells from 4 biological replicates; unpaired Student’s *t*-test). (H) Relative cell proliferation upon MET and NR treatment at 0.1 mM in hAECs (passage 5) using the IncuCyte S3 live cell imaging system (mean ± SD, *n* = 6 biological replicates per group).

The health status of stem cells in various tissues is strongly linked to tissue homeostasis and aging [17–19]; thus we first evaluated the impact of MET and NR on human mesenchymal stem cells (hMSCs), an adult stem cell type presents in most tissues. After drug treatment, we assayed the following senescence-associated features: self-renewal capacity, mitochondrial function, secretion of the pro-inflammatory cytokine interleukin-6 (IL-6), and senescence-associated β-galactosidase (SA-β-Gal) activity. We tested a wide range of concentrations of both MET and NR from 30 μM to 3 mM in Werner syndrome (WS)-specific hMSCs (WS hMSCs; Fig. 1A–1C), a stem cell model of premature aging [20], by an established high-throughput drug screening platform based on the IncuCyte S3 live-cell imaging system [21]. We found that MET promoted self-renewal at concentrations ranging from 30 μM to 3 mM, while NR enhanced self-renewal with the best concentration of 0.1 mM (Fig. 1C). In addition, treatment with MET and NR decreased the percentage of SA-β-Gal-positive cells in WS hMSCs (Fig. 1D). Furthermore, MET and NR attenuated the secretion of IL-6 in WS hMSCs (Fig. 1E), suggesting a decrease in the inflammatory response. In addition, flow cytometry analysis showed that MET and NR treatment improved the mitochondrial membrane potential (MMP) in WS hMSCs (Fig. 1F), indicating their beneficial effect on mitochondrial function. We also tested their effects in other cell types and found that both MET and NR decreased the percentage of SA-β-Gal-positive cells in Cockayne syndrome (CS)-specific hMSCs (CS hMSCs) (Fig. 1G), another human stem cell model of premature aging [22]. Furthermore, we found that MET and NR improved the self-renewal capacity of human arterial endothelial cells (hAECs) (Fig. 1H). Collectively, our results indicate that both MET and NR could mitigate senescence in different human cell types, including aged stem cells, and endothelial cells *in vitro*.

### MET and NR attenuate aging-associated features in diverse rat tissues

**Figure 2.**
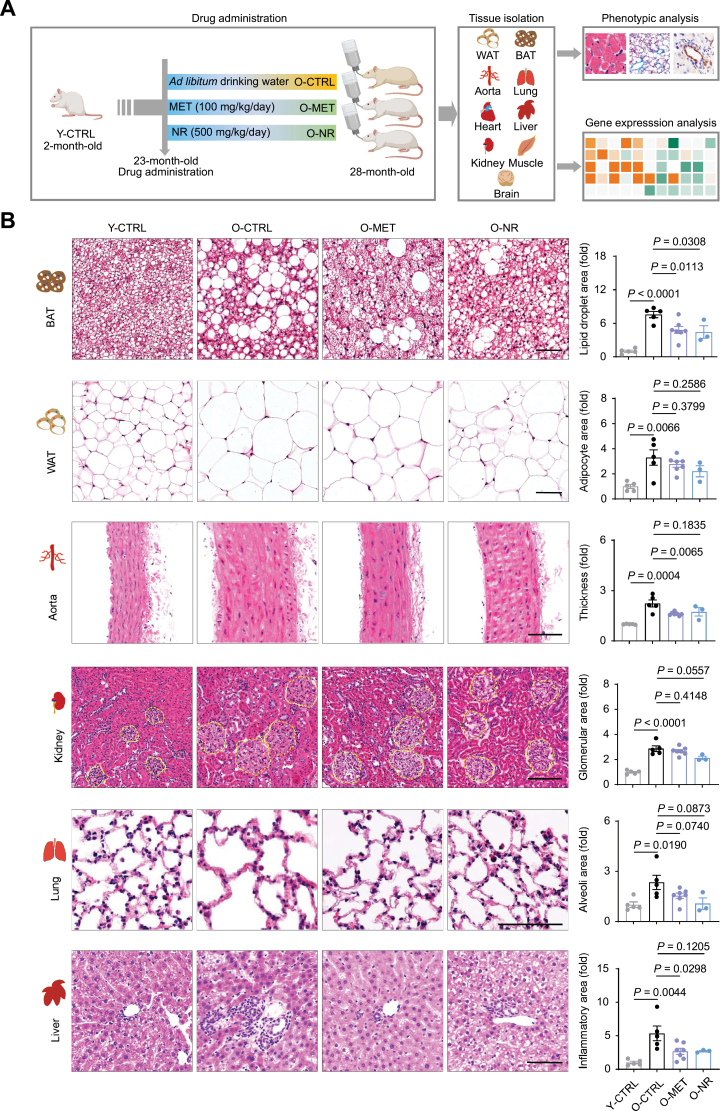
**The geroprotective effects of MET and NR on tissue morphology.** (A) Schematic for the analysis of MET and NR efficacy *in vivo*. 23-month-old rats are treated with *ad libitum* drinking water (O-CTRL), MET (O-MET), or NR (O-NR) for 5 months. Y-CTRL, 2-month-old rats with *ad libitum* drinking water. (B) Representative images (left) and statistics (right) of H&E staining of liver, kidney, lung, BAT, WAT, and aorta from Y-CTRL (*n* = 5), O-CTRL (*n* = 5), O-MET (*n* = 7), and O-NR (*n* = 3) groups (scale bars, 100 μm; mean ± SEM, unpaired Student’s *t*-test). The yellow dotted lines outline glomeruli.

To explore the effects of MET and NR on organismal aging, we treated 23-month-old male *Sprague-Dawley* rats with MET (100 mg/kg) or NR (500 mg/kg) daily in drinking water for 5 months (Fig. 2A). Statistically, we found that MET and NR had no significant effect on rat lifespan, although MET-treated rats showed a tendency to live longer than control ones (*P* value = 0.1027) (Fig. S1A). We further found that the weight of the heart increased during aging and decreased upon MET treatment in aged rats, while no noticeable effect was found in other tissues treated with these drugs (Fig. S1B and S1C).

**Figure 3.**
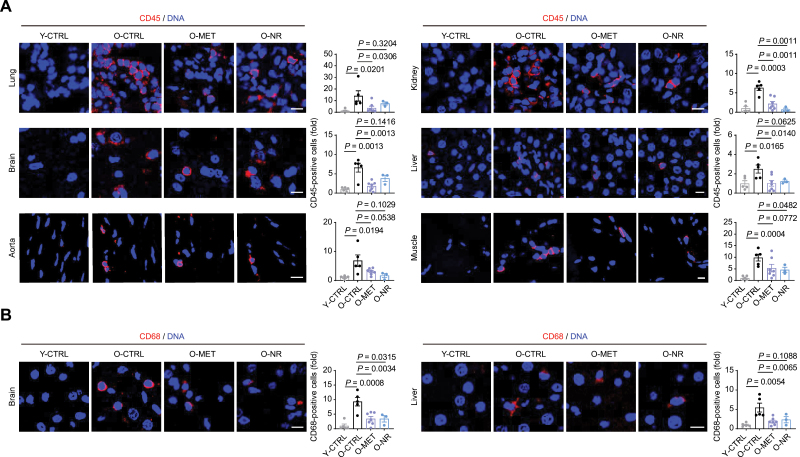
**Both Met and NR reduce inflammation in multiple tissues.** (A) Representative images and statistics of immunostaining of CD45 in lung, kidney, brain, liver, aorta, and muscle of Y-CTRL (*n* = 5), O-CTRL (*n* = 5), O-MET (*n* = 7), and O-NR (*n* = 3) groups (scale bars, 10 μm; mean ± SEM, unpaired Student’s *t*-test). (B) Representative images and statistics of immunostaining of CD68 in brain, and liver of Y-CTRL (*n* = 5), O-CTRL (*n* = 5), O-MET (*n* = 7), and O-NR (*n* = 3) groups (scale bars, 10 μm; mean ± SEM, unpaired Student’s *t*-test).

**Figure 4.**
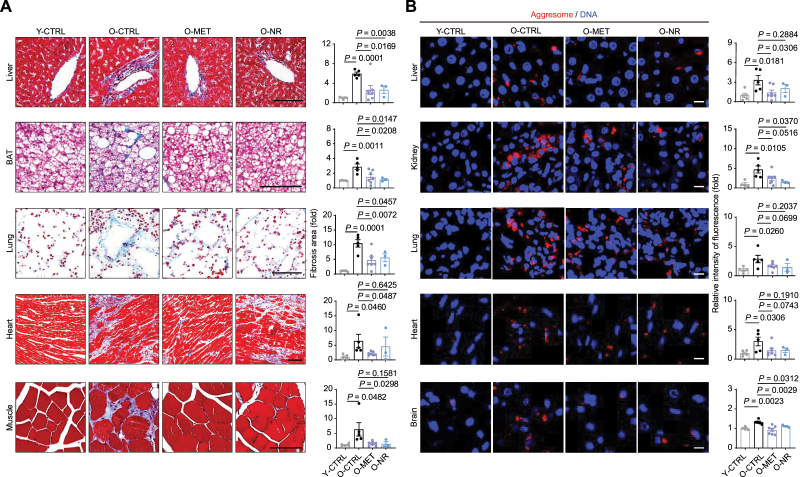
**The geroprotective effects of MET and NR on tissue aging.** (A) Representative images (left) and statistics (right) based on masson staining of liver, BAT, lung, heart, and muscle from Y-CTRL (*n* = 5), O-CTRL (*n* = 5), O-MET (*n* = 7), and O-NR (*n* = 3) groups (scale bars, 100 μm; mean ± SEM, unpaired Student’s *t*-test). (B) Representative images (left) and statistics of fluorescence intensity (right) of aggresome staining of liver, kidney, lung, heart, and brain from Y-CTRL (*n* = 5), O-CTRL (*n* = 5), O-MET (*n* = 7), and O-NR (*n* = 3) groups (scale bars, 10 μm; mean ± SEM, unpaired Student’s *t*-test).

We next explored how MET and NR impact specific tissues [white adipose tissue (WAT), brown adipose tissue (BAT), aorta, lung, kidney, and liver] by classic hematoxylin and eosin (H&E) staining. We noted that the area of BAT lipid droplets in aged rats was several times larger than that of young rats, which was alleviated by both MET and NR (Fig. 2B). A similar (albeit less significant) trend was also found in WAT after treatment with these drugs (Fig. 2B). Together, these findings suggest that MET and NR restore old rats to a lipid metabolic status resembling that of younger animals. In addition, the thickness of arterial vessels increased in aged rats, but this tendency was reduced by both MET and NR to different extents (Fig. 2B). Moreover, the areas of glomeruli in kidney and alveoli in the lung were increased in aged rats, which were repressed by MET and/or NR (Fig. 2B). We also examined inflammatory infiltration, which is upregulated with aging. Tissue-resident CD45-positive cells, representing infiltrating inflammatory cells, increased in aged lung, kidney, brain, liver, aorta, and muscle, but were reduced upon MET or NR treatment (Fig. 3A). CD68 is a marker of macrophage; CD68-positive cells also increased in aged brain and liver, and were reduced upon MET or NR treatment (Fig. 3B). Similar attenuation of inflammatory infiltration was also found in MET- and NR-treated aged liver (Fig. 2B). Next, we examined the effect of MET and NR on tissue fibrosis, which is a major feature of aging and associated with diverse age-related diseases. We found that MET or NR decreased fibrosis in the liver, lung, BAT, heart, and muscle of aged rats (Fig. 4A). Importantly, treatment with MET or NR reduced aggresome formation in several tissues, such as liver, kidney, and brain (Fig. 4B), also indicating the alleviation of tissue senescence. Altogether, these data indicate that MET or NR treatment alleviates aging-associated phenotypes such as inflammation, tissue fibrosis, and protein aggregates in various aged rat tissues.

### MET and NR repress aging-associated gene expression changes

**Figure 5.**
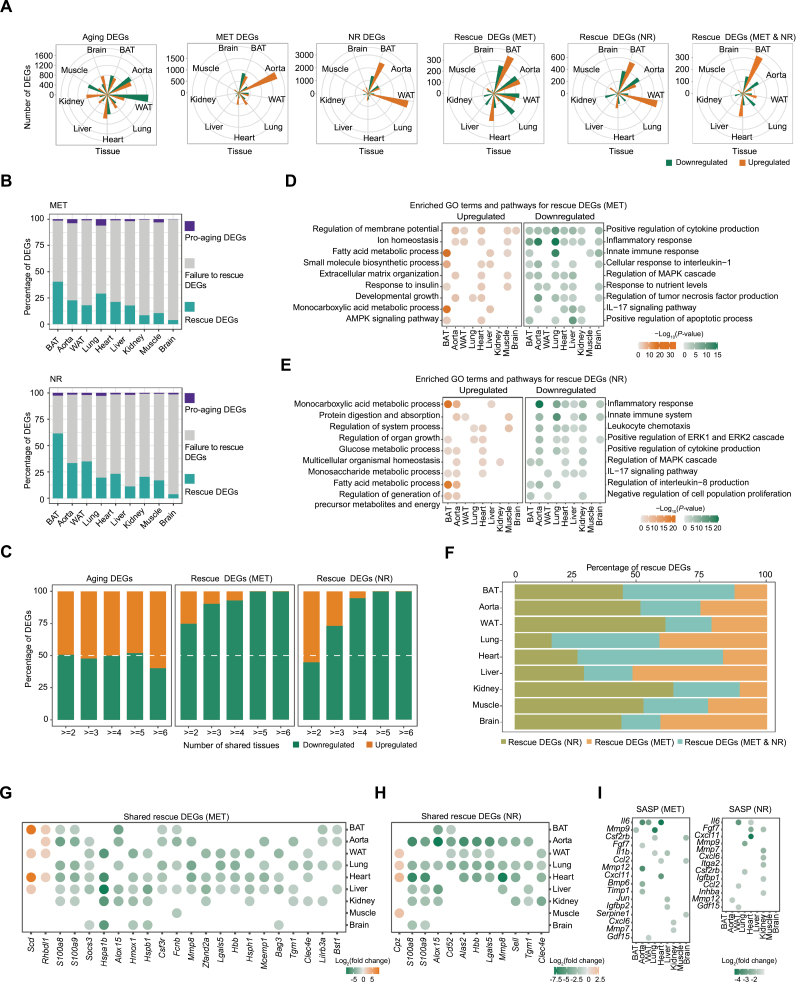
**Changes in the gene expression of different tissues during aging, and with MET or NR treatment.** (A) Rose charts showing the numbers of aging DEGs, MET DEGs, NR DEGs, rescue DEGs (MET), rescue DEGs (NR), and rescue DEGs (MET&NR) in the nine tissues (orange, upregulated; green, downregulated). (B) Percentages of rescue DEGs (green), failure to rescue DEGs (gray), and pro-aging DEGs (purple) in the nine tissues with MET or NR treatment. (C) Percentages of shared aging DEGs, rescue DEGs (MET) and rescue DEGs (NR) across tissues (orange, upregulated; green, downregulated). (D and E) Representative GO terms and pathways enriched in rescue DEGs shared by at least two tissues upon MET (D) or NR (E) treatment. The color keys from light gray to orange or green indicate -log_10_(*P*-value) from low to high. (F) Percentages of rescue DEGs (MET), rescue DEGs (NR), and rescue DEGs (MET&NR) in the nine tissues. (G and H) Point plots showing the upregulated and downregulated rescue DEGs shared by at least four tissues upon MET (G) or NR (H) treatment. The color key from green to orange indicates log_2_(fold change) from low to high. (I) Point plots showing the downregulated SASP-related DEGs across tissues upon MET (left) or NR (right) treatment. The color key from green to gray indicates log_2_(fold change) from low to high.

To further uncover the effects of both MET and NR systematically, we collected nine tissues (BAT, WAT, aorta, lung, kidney, liver, heart, muscle, and brain) from MET- or NR-treated and control old groups as well as a 2-month-old young group, and subjected them to bulk RNA sequencing (Fig. 2A). We identified aging-related differentially expressed genes (aging DEGs) in different tissues by comparing the transcriptomes of young and old control rats (Fig. 5A and 5B, Fig. S2A–S2C). By evaluating the transcriptomes from MET- or NR- treated and control old rats, we defined MET-induced DEGs and NR-induced DEGs across multiple tissues as MET DEGs or NR DEGs (Fig. 5A). According to our DEG analysis, the transcriptomes of WAT, aorta, and heart appeared more likely to be affected by aging (Fig. 5A). Based on the numbers of MET DEGs and NR DEGs, BAT, WAT, and aorta were more responsive to both MET and NR treatment (Fig. 5A). Next, we explored the potential effects of MET or NR on aging-associated genes by cross-referencing the aging DEGs to MET DEGs or NR DEGs. We identified aging DEGs whose expression was at least partially reversed (rescued) by MET or NR as “MET Rescue DEGs” or “NR Rescue DEGs” (Fig. 5A and 5B). Consistent with the geroprotective effects of MET and NR on aged tissue transcript levels shown above, both MET and NR treatment diminished the numbers of aging DEGs in multiple tissues (Fig. 5A and 5B, Fig. S2A and S2B), i.e, >18% of aging DEGs were rescued in about half of the aged tissues (e.g., BAT, lung, and aorta) treated with MET or tissues (e.g., BAT, WAT, and aorta) treated with NR (Fig. 5A and 5B, Fig. S2A and S2B). We found that, for the indicated treatment (MET or NR), the percentage of rescued aging DEGs varied depending on the tissue type (Fig. 5A and 5B, Fig. S2A and S2B). Among the nine tissues analyzed, adipose tissue (BAT and WAT) and aorta displayed the most extensive changes in percentage and/or the number of aging DEGs reversed by MET and NR (Fig. 5A and 5B, Fig. S2A and S2B). This is consistent with the previous reports that MET and NR exert geroprotective effects by targeting metabolism [12, 23]; adipose tissue is a major player that maintains homeostasis of physiological metabolism [24]; and aorta is the key tissue responsible for metabolite transport via the blood. Overall, these findings suggest that MET and NR reverse changes in the expression of a substantial percentage of aging-associated genes, and display tissue-specific modulation, with adipose tissue and the aorta being the top target tissues of MET and NR.

To reveal common changes in gene expression caused by MET or NR treatment across tissues, we further analyzed the MET or NR rescue DEGs shared by more than one tissue (tissue-shared MET or NR rescue DEGs). As shown in Fig. 5C, about half of the tissue-shared aging DEGs were upregulated, comparable to that of downregulated DEGs. However, such a generally symmetrical distribution pattern was not shown in tissue-shared MET or NR rescue DEGs. Both MET and NR preferred to reverse (rescue) the tissue-shared DEGs that were upregulated (not downregulated) during the aging process (Fig. 5C). Notably, Gene Ontology (GO) analysis demonstrated that both MET and NR repressed the expression of genes enriched in signaling pathways such as the inflammatory response and positive regulation of cytokine production across different tissues (Fig. 5D and 5E, Fig. S2D; elaborated further below). Altogether, our data suggest that MET and NR may utilize a common systemic mechanism that reverses aging-related gene expression in multiple tissues.

### MET and NR regulate diverse molecular pathways in aged tissues

We further analyzed the biological processes and functions affected by MET and NR. As shown by GO analysis, genes associated with the inflammatory response, response to nutrient levels, positive regulation of cytokine production, and positive regulation of apoptotic process increased during aging and were downregulated in at least four tissues upon MET administration (Fig. 5D, Fig. S2D). In contrast, the inflammatory response, positive regulation of cytokine production, and regulation of the mitogen-activated protein kinase cascade was decreased in at least four tissues upon NR administration (Fig. 5E, Fig. S2D). Moreover, gene set enrichment analysis (GSEA) confirmed that MET and NR attenuated the expression of genes involved in the interferon-α response in multiple aged tissues (Fig. S2E), further confirming their common anti-inflammatory impact.

Conversely, we found that genes in the extracellular matrix organization, fatty acid metabolic process, small molecule biosynthetic process, and developmental growth were reduced during aging and upregulated in at least three tissues upon MET treatment (Fig. 5D, Fig. S2D). Meanwhile, as for NR treatment, the signaling pathways, including protein digestion and absorption, monocarboxylic acid metabolic process, regulation of organ growth, and multicellular organismal homeostasis, were upregulated in at least three tissues (Fig. 5E, Fig. S2D). Notably, both MET and NR enhanced fatty acid metabolism, consistent with adipose tissue being one of the top target tissues affected by MET and NR (Fig. 5A, 5B, 5D, 5E).

In addition, we applied integrative comparative analysis with aging/longevity-associated genes from the Aging Atlas database [25], and found that the expression of aging-associated genes such as *Hspa1b* and *Top2a* were modulated by MET or NR in different tissues (Fig. S2F). We further applied transcription factor (TF) enrichment analysis using the R package RcisTarget and identified “Rescue TFs” that were enriched for aging and MET/NR DEGs across different tissues (Fig. S3A–S3C). Both MET and NR inactivated many TFs associated with inflammation, including *Cebpb*, *Rela*, and *Stat2*. Lastly, we found that only a minimal percentage of pro-aging DEGs were induced by MET or NR treatment (Fig. S3D). Together with undetected side effects at the histological level, our results indicated that MET/NR treatment may elicit controllable side effects even after long-term treatment. Collectively, these data show that both MET and NR enhance biological processes such as fatty-acid metabolism and depress the inflammatory response in aged tissues.

### MET and NR elicit common and specific changes in gene expression in aged rats

**Figure 6.**
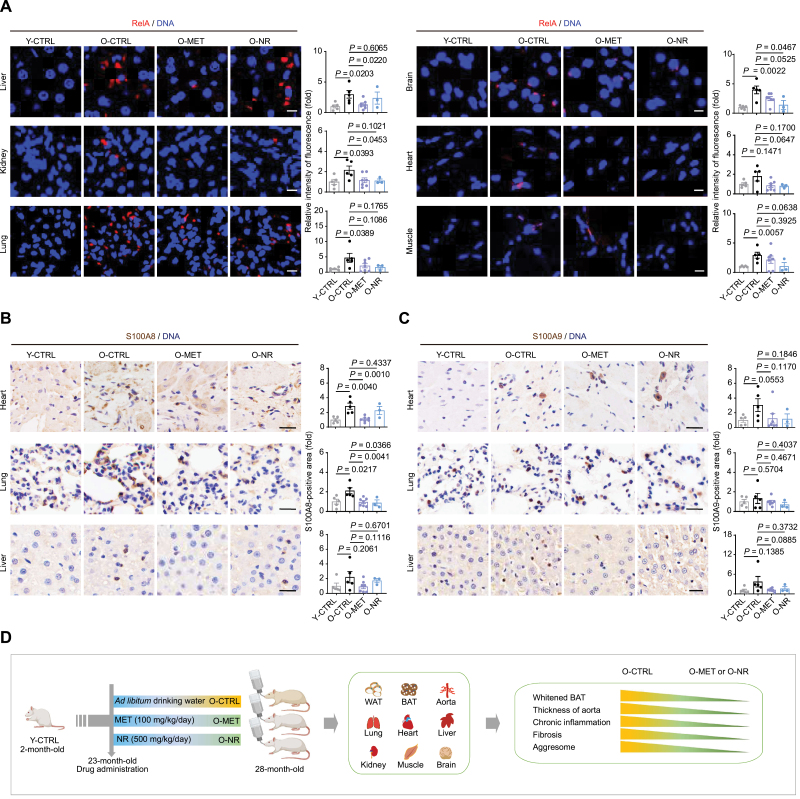
**MET and NR repress systemic inflammation in the indicated tissues.** (A) Representative images and statistics of immunostaining of RelA in liver, kidney, lung, brain, heart, and muscle of Y-CTRL (*n* = 5), O-CTRL (*n* = 5), O-MET (*n* = 7), and O-NR (*n* = 3) groups (scale bars, 10 μm; mean ± SEM, unpaired Student’s *t*-test). (B and C) Representative images and statistics of immunohistochemical staining of S100A8 (B) and S100A9 (C) in heart, lung, and liver of Y-CTRL (*n* = 5), O-CTRL (*n* = 5), O-MET (*n* = 7), and O-NR (*n* = 3) groups (scale bars, 20 μm; mean ± SEM, unpaired Student’s *t*-test). (D) Schematic diagram illustrating the geroprotective effects of MET or NR in aged rat tissues.

Next, we applied a comprehensive comparative analysis to investigate the similarities and differences in the effects of MET and NR on changing gene expression in aged rat tissues. As shown in Fig. S4A, MET DEGs partially overlapped with NR DEGs in each type of tissue. Similarly, MET rescue DEGs partially overlapped with NR rescue DEGs for each tissue (Fig. 5F). We found that over a quarter of the rescue DEGs in 5/9 tissues were common upon both MET and NR treatment (Fig. 5F, Fig. S4A–S4C). Of note, both MET and NR treatment commonly upregulated DEGs across tissues associated with fatty-acid biosynthesis and proteolysis, such as *Scd* and *Rhbdl1* (Fig. S4D). By contrast, the DEGs downregulated by both MET and NR across tissues were mainly associated with the inflammatory response, and included *S100a8, S100a9*, and *Mmp8* (Fig. 5G and 5H, Fig. S2C), consistent with the anti-inflammatory effect of MET and NR. We also found that genes associated with the senescence-associated secretory phenotype (SASP), like *Il6*, *Ccl2*, *Fgf7*, *Mmp9,* and *Mmp12*, were reduced by MET and NR treatment (Fig. 5I). Consistent with this, the main regulator of SASP, RelA, was found in the list of rescue TFs downregulated by both MET and NR (Fig. S3C). To confirm their expression pattern at the protein level, we investigated the inflammation-associated genes *RelA*, *S100a8*, and *S100a9*, all of which were downregulated by MET or NR treatment based on our RNA-seq data. By immunostaining, we found that MET or NR reduced (or showed a tendency to reduce) RelA protein expression in the liver, kidney, lung, brain, heart, and muscle of aged rats compared with controls (Fig. 6A), in agreement with the repressive influence of pro-inflammatory pathway by these two compounds (Fig. S3C). In addition, we found that the protein levels of the pro-inflammatory factors S100A8 and S100A9 increased during aging, which were rescued upon MET or NR treatment in various tissues (Fig. 6B and 6C). Since both *S100a8* and *S100a9* were upregulated in most (8/9) aged tissues analyzed (Fig. S2C), these genes might be used as aging fingerprints across multiple tissues for future aging-related studies, which is also consistent with the reported role of S100A8 and S100A9 in aging and aging-related diseases [5, 23, 24, 26]. Taken together, these data demonstrate that MET and NR regulate a common set of inflammation-related genes in aged tissues that may be core mechanisms underlying the geroprotective effect of these compounds.

## Discussion

In this study, we systemically conducted histomorphological and transcriptome analyses to delineate aging and the effects of MET and NR in nine tissues of aged rats. We found that both MET and NR alleviated the aging-associated phenotypes, especially systemic inflammation, tissue fibrosis, and aggresome formation in aged rat tissues. We further showed that both MET and NR partially rescued aging DEGs in several tissues, and the most remarkable effects were found in adipose and aortic tissues. Next, we also demonstrated that MET and NR both had drug-specific and common effects in rescuing aging gene expression and modulating the biological signaling pathways in aged rats. Collectively, this work provides a wealth of molecular information about both aging and the effects of pharmacological intervention with MET or NR.

The beneficial effects of MET and NR in tissues have been shown in many studies, but most of them focused on a single or a few tissues. For example, MET has been shown to have a beneficial effect on adipose tissue [27–29], blood vessels [30, 31], liver [32, 33], and heart [34], while NR has a protective effect in BAT [35], aorta [26], and muscle [36, 37]. Despite these reports, the impact of both drugs at a systemic level across multiple tissues remained unclear. Here, we investigated the effects of both drugs in nine major aged tissues at different levels, and comprehensively compared the two chemicals. We found that, at the transcriptional level, aged tissues responded to both MET and NR in both tissue-specific and non-tissue-specific manners, with adipose tissue and the aorta being the top target tissues. Accordingly, at the histomorphological level, we found that both MET and NR decreased (or tended to reduce) BAT whitening, WAT cell size, aortic wall thickness, and alveolar area, confirming a protective effect. We also noticed that MET and NR are specific in terms of certain evaluation indicators for the geroprotective effect. For instance, MET showed the ability to reduce cardiac fibrosis, while NR did not; conversely, NR tended to reduce the glomerular area in the kidney, while MET did not. Moreover, we showed that inflammation-related genes and signaling pathways are repressed by both MET and NR, consistent with the notion that chronic inflammation is a major deleterious factor for healthspan [38].

In this study, we displayed that MET and NR improved various aspects of healthspan in rats but did not statistically alter their lifespan, although MET has been shown to extend lifespan in nematodes and mice [16, 39]. Consistent with our results, a previous study also showed MET did not extend lifespan in rats [40]. Thus, the lifespan extension effect of MET might be species-specific. Additionally, in a study of MET testing in mice [16], 11-month-old male C57BL/6 mice were given 0.1% MET (w/w) in the diet, which resulted in a 5.83% increase in mean lifespan. The dosing regimen, such as dose, duration, and initiation time point, was different from that employed in our rat study, which may also contribute to the differences in lifespan regulation. While NR was reported to elongate healthspan and lifespan in mice [11], its effect on the lifespan of rats has not been evaluated so far. Here, we showed that NR alleviated the aging of various tissues in rats but did not extend their lifespan. Therefore, the regulation of lifespan by NR across different mammalian species requires more research to elucidate.

So far, both MET and NR have been demonstrated to have a protective effect against aging-related diseases. Patients with type 2 diabetes undergoing MET monotherapy survive longer than matched, non-diabetic controls [41]. Meanwhile, chronic NR supplementation is well-tolerated and provides initial insight into the effects on physiological function, particularly the potential benefits of NR for reducing blood pressure and arterial stiffness [26]. In rats, our data pointed to the notion that MET and NR reduced fibrosis and inflammation in the heart and muscle, and decreased vascular wall thickness, further supporting the geroprotective effects of both drugs. As an indirect evidence, we found oncogenes like *Myc* and *Stat* were downregulated after MET and NR treatment, suggesting that MET and NR have minimal tumor-promoting risk (even have certain anti-cancer properties), at least in the rat.

In summary, our results demonstrate a geroprotective role of both MET and NR on senescent human cells and aged rat tissues and offer a systematic multi-tissue molecular pathway resource for investigating the effects of MET and NR on aged rats. These findings may lay a foundation for further understanding and combating aging-related disorders.

## Research limitations

Although this study suggests a potential geroprotective effect of MET and NR in rats, this study also has certain limitations. First, only male rats were employed in this study, so the results of this study may be limited by species and gender. Second, we treated aged rats (23 months old) with fixed concentrations of MET (100 mg/kg) or NR (500 mg/kg) for 5 months. To explore the maximal geroprotective effect of these two drugs, more experimental conditions (e.g., different dosing regimens, starting time points, and varied durations) are required to take into consideration in future studies. Third, studies using larger animal populations are needed to evaluate the lifespan-extending effects of MET and NR, as well as other compounds. Likewise, RNA-seq analysis that considers individual differences will deepen our understanding of the effects of these drugs on gene expression. Finally, to better understand the safety and efficacy of MET and NR in promoting health in the elderly, periclinal experiments based on non-human primates should be considered in the future.

## Materials and methods

### Cell culture

Human embryonic stem cell-derived WS hMSCs and human induced pluripotent stem cell-derived CS hMSCs were cultured on gelatin-coated plates in MSC medium containing α-MEM with GlutaMAX (Gibco), 10% fetal bovine serum (Gibco, Cat#10099-141), 1% penicillin/streptomycin (Gibco), 0.1 mM non-essential amino-acids (Gibco), and 1 ng/mL fibroblast growth factor 2 (Joint Protein Central, Cat#BBI-EXP-002). hAECs (Lonza, CC-2535) were maintained on collagen-coated plates in endothelial cell growth 2 medium (Lonza, CC-3162) with 1% penicillin/streptomycin (Gibco). No mycoplasma contamination was detected during cell culture. MET hydrochloride (Tocris, 2864) and NR chloride (Selleck, S2935) were used in cell models.

### Experimental animals

Wild-type male *Sprague-Dawley* rats were from the Beijing Vital River Laboratory Animal Technology Co., Ltd. At the age of 23 months, the rats were randomly divided into O-CTRL, O-MET, and O-NR groups in a specific-pathogen-free-grade facility with individually ventilated cages. The drugs were administered in drinking water for 5 months. Niagen™, a synthetic form of NR, was used at an oral dose of 500 mg/kg/d. Glucophage^®^, a MET hydrochloride tablet, was used at an oral dose of 100 mg/kg/d. The water bottles were kept out of light and changed every 2 days. Young rats (2 months old) drinking normal water (Y-CTRL group) were used as controls. The room was maintained at a controlled temperature (20°C–25°C), humidity (30%–70%), and light exposure (12-h light-dark cycle). All experimental procedures were approved by the Chinese Academy of Sciences Institutional Animal Care and Use Committee.

### Tissue dissociation

Tissues from randomly selected 2-month-old Y-CTRL, 28-month-old O-CTRL, O-MET, and O-NR animals were isolated after chloral hydrate anesthesia and systemic perfusion with normal saline. The same tissue samples of the same size from each group were washed with cold PBS and quickly frozen in liquid nitrogen for DNA, RNA, and protein extraction; histopathological and histochemical samples were fixed in 4% paraformaldehyde (PFA). The samples were kept on ice during the entire separation process.

### H&E staining

H&E staining was performed as previously described [42, 43]. Paraffin-embedded tissues were sectioned at 5 μm on a rotary microtome. The sections were mounted onto glass microscope slides, dried at 56°C for 24 h, and stored at room temperature. For H&E staining, the sections were first deparaffinized in xylene, and rehydrated in a gradient of alcohols (100%, 100%, 95%, 90%, 80%, 70%, and 50%) and then briefly washed in distilled water. The sections were then incubated in hematoxylin solution until the desired degree of staining was achieved (Servicebio, China), and washed in running tap water to remove excess hematoxylin. The sections were then differentiated in 1% acid alcohol for 1–2 s, and rinsed in running tap water for 1 min. The sections were then stained with eosin to the desired shade of pink, and dehydrated in a graded series of alcohols and xylene. Finally, the sections were mounted with Cytoseal-60 (Stephens Scientific).

### Masson’s trichrome staining

Masson’s trichrome staining was performed as previously published [44]. Paraffin-embedded tissue sections were deparaffinized in 100% xylene and rehydrated in a graded series of alcohols (100%, 95%, 90%, 80%, and 70%). After washing in distilled water for 5 min, sections were stained overnight with potassium bichromate and rinsed in running water for 5–10 min. The sections were then stained with Weigert’s iron hematoxylin solution for 10 min, followed by another rinse in running warm water for 10 min. Next, the sections were stained with Ponceau–acid fuchsin for 5–10 min, washed in distilled water, and differentiated in phosphomolybdic–phosphotungstic acid for 10–15 min, followed by direct transfer (without rinsing) to aniline blue solution for 5–10 min; after a brief rinse in distilled water, the sections were differentiated in 1% acetic acid for 2–5 min. The sections were washed 3 times in distilled water for 5 min each, dehydrated in an ethanol gradient (70%, 80%, 90%, 100%, and 100%), and 100% xylene, and mounted in resinous mounting medium.

### SA-β-Gal staining

SA-β-Gal staining followed a previously published protocol [45–47]. In brief, harvested cells were fixed in 2% formaldehyde and 0.2% glutaraldehyde at room temperature for 5 min, and were then incubated with freshly prepared SA-β-Gal staining solution overnight at 37°C (X-gal powder was from Amresco; and the other reagents were from Sigma). Images were captured and the percentages of SA-β-Gal-positive cells were calculated using ImageJ software (version: 1.8.0).

### Enzyme-linked immunosorbent assay (ELISA)

IL-6 levels in the supernatant of WS hMSCs were measured using a commercially available ELISA kit (Biolegend, 430504) according to the manufacturer’s instructions.

### MMP analysis

MMP was assessed with the Cell Meter™ JC-10 kit (22801, AAT Bioquest). MMP levels were quantified using specific fluorescence intensities at Ex/Em = 490/590 nm (JC-10 aggregate emission) and 490/530 nm (JC-10 monomer emission) (FL590/FL530). The analysis was performed on the LSR Fortessa cell analyzer (BD, USA), and the data were further analyzed using FlowJo software (TreeStar, Ashland, OR).

### Immunofluorescence staining

Immunofluorescence staining was applied as previously published [48]. Paraffin-embedded sections were deparaffinized in 100% xylene three times for 10 min each, and rehydrated through an alcohol gradient (100%, 100%, 95%, 90%, 80%, 70%, and 50%). Then, the sections were rinsed in distilled water, and microwaved in 10 mM sodium citrate buffer (pH 6.0) for 20 min. Upon cooling to room temperature, the sections were rinsed in PBS, and permeabilized with 0.4% Triton X-100 in PBS for 1 h at room temperature, followed by rinsing three times in PBS for 5 min each. The sections were blocked with blocking buffer (10% donkey serum in PBS) for 1 h at room temperature, followed by incubation with primary antibodies at 4°C overnight. The sections were rinsed three times in PBS for 10 min for each, and then incubated with fluorescence-labeled secondary antibodies at room temperature for 1 h. Nuclei were counterstained with Hoechst 33342 (Thermo Fisher Scientific). Images were acquired with a Zeiss LSM900 laser scanning confocal microscope.

The following primary antibodies were used for immunofluorescence staining: anti-RelA (8242s, 1:100) from Cell Signaling Technology, and anti-CD45 (ab10558, 1:200), anti-CD68 (ab125212, 1:100) from Abcam.

### Immunohistochemical staining

As previously described [49], paraffin-embedded rat tissue sections were deparaffinized and rehydrated using 100% xylene and an alcohol gradient, followed by a heat-mediated antigen retrieval procedure by microwaving in 10 mM sodium citrate buffer (pH 6.0) for 20 min and permeabilization with 0.4% Triton X-100 for 1 h at room temperature. Endogenous peroxidases in the sections were treated with 3% H_2_O_2_ for 10 min and then blocked with 10% donkey serum in PBS for 1 h. The sections were incubated with the primary antibodies overnight. The next day, the sections were washed 5 times with PBS. Samples were incubated with HRP-conjugated secondary antibodies (ZSGB-BIO) for 1 h and visualized using the DAB Substrate kit (ZSGB-BIO) and counterstained with hematoxylin. Images were acquired on an Olympus microscope.

The following primary antibodies were used for immunohistochemical staining: anti-S100A8 (Abcam, ab180735, 1:100) and anti-S100A9 (Abcam, ab22506, 1:100).

### Aggresome staining

Aggresomes were stained as previously published [50]. In brief, frozen tissue sections were fixed with 4% PFA in PBS for 15 min, and then permeabilized with 0.4% Triton X-100 in PBS for 30 min, incubated with the Proteostat^®^ aggresome detection kit (Enzo, ENZ-51035-K100) at 1:3000 dilution in PBS for 3 min, and then de-stained in 1% acetic acid for 20 min. After washing three times with PBS for 10 min each, the sections were counterstained with Hoechst 33342 (Thermo Fisher Scientific), and then washed three times with PBS for 10 min each. Lastly, the sections were mounted with Cytoseal-60 (Stephens Scientific). Images were captured using a Zeiss LSM900 laser scanning confocal microscope and data were analyzed using ImageJ.

### Western blot analysis

Cells were lysed with 2 × SDS buffer (Sigma-Aldrich), and then the BCA kit (Dingguochangsheng Biotechnology, BCA-02) was used to measure protein concentrations. For Western blot detection, 20 μg protein lysate per sample was separated by SDS-PAGE electrophoresis and transferred onto PVDF membranes (Millipore). The membranes were blocked with 5% milk (BBI Life Sciences), and incubated with specific primary antibodies overnight at 4°C and the relevant HRP-conjugated secondary antibody at room temperature for 1 h. Blots were captured using Image Lab software (ChemiDoc XRS+ system, Bio-Rad) and quantified with ImageJ (version: 1.8.0).

The primary antibody anti-WS helicase WRN (Ab200, 1:500) from Abcam was used for Western blot.

### RNA-seq library construction and sequencing

Total RNA from each rat tissue was isolated using TRIzol (15596018, Gibco). After total RNA extraction, equal amounts of RNA from each individual were pooled together and subjected to three technical repeats for sequencing using the NEBNext^®^ Poly (A) mRNA Magnetic Isolation Module. To construct sequencing libraries, we used the NEBNext^®^ Ultra™ RNA Library Prep Kit for Illumina (NEB, USA) following the manufacturer’s protocol, and the generated libraries were sequenced on Illumina platforms with paired-end 150-bp sequencing. Quality control and sequencing were all obtained using Novo-gene Bioinformatics Technology.

### RNA-seq data processing

Raw data were trimmed using Trim Galore software (version 0.4.5) (github.com/FelixKrueger/TrimGalore) to remove reads of low quality or with adapters. The cleaned reads were then mapped to the UCSC rat (*Rattus norvegicus*) reference genome (rn6) using HISAT2 (version 2.2.1) [51]. The expression levels of each annotated gene were calculated using HTSeq (version 0.12.4) [52]. DEGs were calculated using the R package DESeq2 (version 1.32.0) with a cutoff Benjamini-Hochberg adjusted *P*-value < 0.05 and absolute log_2_ (fold change) > 1.5 [53].

### GO and gene set analysis

GO biological process and pathway enrichment were analyzed using Metascape (metascape.org) [54], and the results were visualized with the R package ggplot2 (version 3.3.5). Gene set enrichment was conducted using GSEA (version 4.1.0) with default parameters [55]. The aging-associated genes were from the Aging Atlas (bigd.big.ac.cn/aging/index).

### TF enrichment analysis

TF enrichment was analyzed using the R package RcisTarget (version 1.12.0).

### Statistical analysis

Data are presented as the mean ± SEM. Statistical analyses were performed using two-tailed Student’s *t*-test, one-way ANOVA, or Log-rank test as appropriate in GraphPad PRISM software (v9). *P* values < 0.05 were considered statistically significant.

## Supplementary Material

lnac045_suppl_Supplementary_Material

## Data Availability

High-throughput sequencing data generated in this study have been deposited in the Genome Sequence Archive database with accession number CRA006823.
