# Supplementary material for: A comparative study of metformin and nicotinamide riboside in alleviating tissue aging in rats
Source: Life Med. 2022 Oct 26;2(1):lnac045. doi: 10.1093/lifemedi/lnac045 (PMC11749560; doi:10.1093/lifemedi/lnac045)
Supplement: lnac045_suppl_Supplementary_Material [file lnac045_suppl_Supplementary_Material.pdf]

Supplementary Information for "A comparative study of metformin and nicotinamide riboside in alleviating tissue aging in rats"

Supplemental Figures

Figure S1. Lifespan and tissue weights in MET- and NR-treated rats.

(A) Survival curves of O-CTRL (n = 12), O-MET (n = 7), and O-NR rats (n = 6) (Log-rank test).

(B) Organ weights of lung, liver, heart, brain, and kidney in Y-CTRL (n = 5), O-CTRL (n = 5), O-MET (n = 7), and O-NR (n = 3) groups (mean ± SEM, unpaired Student's t-test).

(C) Relative organ weights of lung, liver, heart, brain, and kidney in Y-CTRL (n = 5), O-CTRL (n = 5), O-MET (n = 7), and O-NR (n = 3) groups (mean ± SEM, unpaired Student's t-test).

Figure S2. Changes in the transcriptional profiles of different tissues during aging, MET, and NR treatment.

(A) Heatmaps showing the distribution of rescue DEGs (green), failure to rescue DEGs (orange), MET- or NR-specific DEGs (dark blue), and pro-aging DEGs (pink) in the nine tissues. Each column represents one gene. Orange, upregulated [$\log_2$(fold change) > 1.5, adjusted P-value < 0.05]; green, downregulated [$\log_2$(fold change) < -1.5, adjusted P-value < 0.05]; light grey, unchanged [|$\log_2$(fold change)| ⩽ 1.5 or adjusted P-value ⩾ 0.05].

(B) Rose charts showing the numbers of MET or NR pro-aging DEGs, and MET- or NR-specific rescue DEGs in the nine tissues (orange, upregulated; green, downregulated).

(C) Point plots showing the aging DEGs, MET DEGs, and NR DEGs shared by at least five tissues. The color keys from light grey to orange or green indicate –$\log_{10}$(P-value) from low to high.

(D) Representative GO terms and pathways enriched in aging DEGs shared by at least four tissues. The color keys from light grey to orange or green indicate –$\log_{10}$(P-value) from low to high.

(E) Gene set enrichment analysis plots showing a decreased interferon-α response in different tissues with MET or NR treatment.

(F) Heatmap showing the genes shared between rescue DEGs and aging-associated genes in the Aging Atlas database among nine tissues. The color key from green to orange indicates $\log_2$(fold change) from low to high.

Figure S3. Changes in the transcriptional regulatory networks and side-effects of different tissues upon MET and NR treatment.

(A) Rose charts showing the numbers of aging TFs, MET or NR TFs, and MET or NR rescue TFs, in the nine tissues (orange, upregulated; green, downregulated).

(B, C) Point plots showing the upregulated (orange) and downregulated (green) MET rescue TFs, NR rescue TFs, and common rescue TFs shared by at least two tissues upon MET or NR treatment.

(D) Pie charts showing the percentages of pro-aging DEGs upon MET or NR treatment in the nine tissues.

Figure S4. Changes in gene expression in response to MET and NR treatment.

(A) Heatmaps showing common rescue DEGs upon MET and NR treatment in the nine tissues. The color key from green to orange indicates z-score from low to high.

(B) Venn diagrams showing the numbers of common rescue DEGs upon both MET and NR treatment in the nine tissues.

(C) Representative GO terms and pathways enriched in common rescue DEGs shared by at least two tissues upon both MET and NR treatment. The color keys from light grey to orange or green indicate $-\log_{10}(P\text{-value})$ from low to high.

(D) Heatmap showing the common rescue DEGs shared by at least three tissues upon both MET and NR treatment. The color key from green to orange indicates $\log_2$(fold change) from low to high.

Figure S1

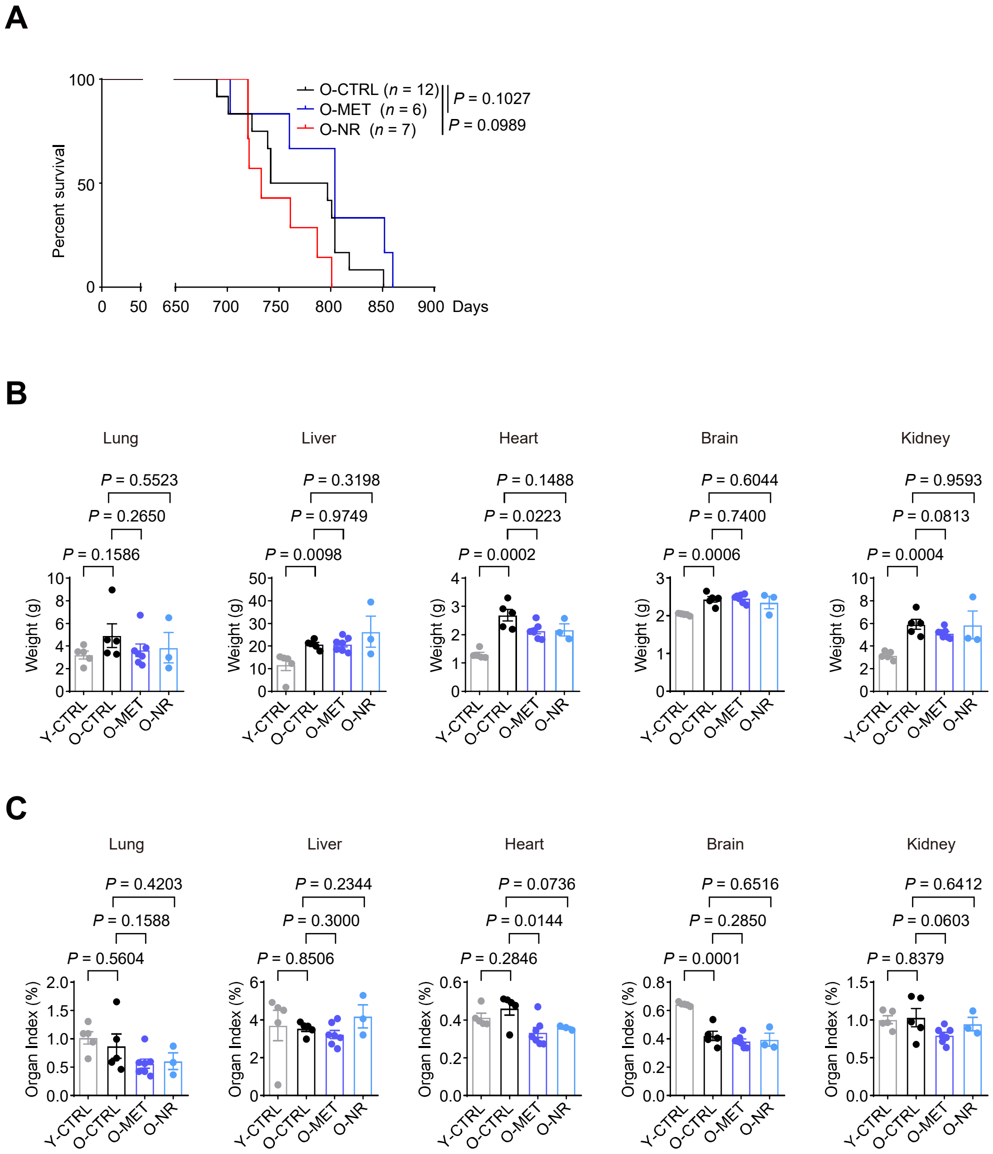
A
B
C
Percent survival
Days
O-CTRL (n = 12)
O-MET (n = 6)
O-NR (n = 7)
P = 0.1027
P = 0.0989
Lung
Liver
Heart
Brain
Kidney
Weight (g)
Organ Index (%)
Y-CTRL
O-CTRL
O-MET
O-NR
P = 0.5523
P = 0.2650
P = 0.1586
P = 0.3198
P = 0.9749
P = 0.0098
P = 0.1488
P = 0.0223
P = 0.0002
P = 0.6044
P = 0.7400
P = 0.0006
P = 0.9593
P = 0.0813
P = 0.0004
P = 0.4203
P = 0.1588
P = 0.5604
P = 0.2344
P = 0.3000
P = 0.8506
P = 0.0736
P = 0.0144
P = 0.2846
P = 0.6516
P = 0.2850
P = 0.0001
P = 0.6412
P = 0.0603
P = 0.8379

Figure S2

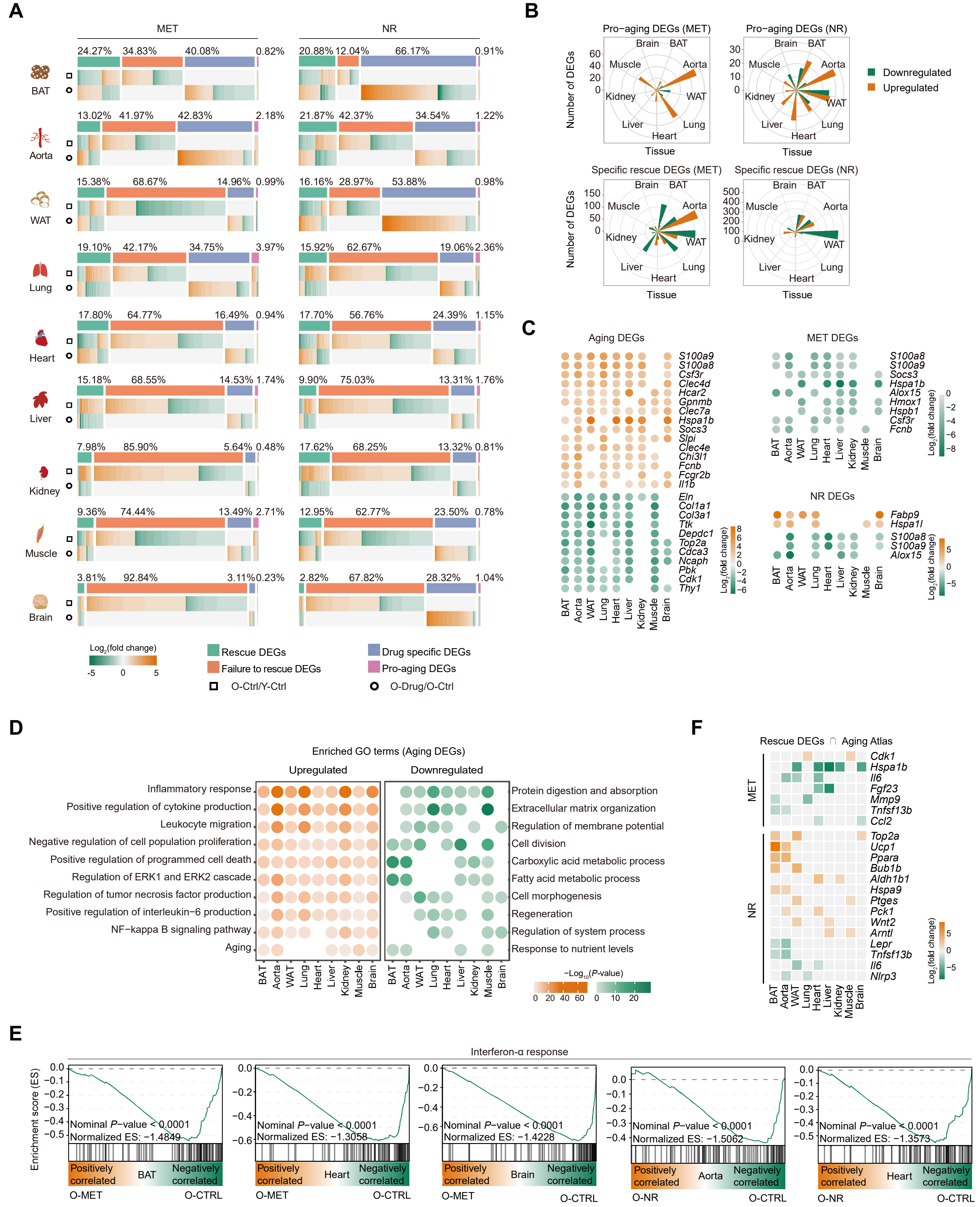

A
MET
NR
BAT
Aorta
WAT
Lung
Heart
Liver
Kidney
Muscle
Brain
Log2(fold change)
Rescue DEGs
Failure to rescue DEGs
Drug specific DEGs
Pro-aging DEGs
O-Ctrl/Y-Ctrl
O-Drug/O-Ctrl
B
Pro-aging DEGs (MET)
Pro-aging DEGs (NR)
Specific rescue DEGs (MET)
Specific rescue DEGs (NR)
Number of DEGs
Tissue
Downregulated
Upregulated
C
Aging DEGs
MET DEGs
NR DEGs
D
Enriched GO terms (Aging DEGs)
Upregulated
Downregulated
Inflammatory response
Positive regulation of cytokine production
Leukocyte migration
Negative regulation of cell population proliferation
Positive regulation of programmed cell death
Regulation of ERK1 and ERK2 cascade
Regulation of tumor necrosis factor production
Positive regulation of interleukin−6 production
NF−kappa B signaling pathway
Aging
Protein digestion and absorption
Extracellular matrix organization
Regulation of membrane potential
Cell division
Carboxylic acid metabolic process
Fatty acid metabolic process
Cell morphogenesis
Regeneration
Regulation of system process
Response to nutrient levels
−Log10(P-value)
E
Interferon-α response
Enrichment score (ES)
Nominal P−value < 0.0001
Normalized ES: −1.4849
Normalized ES: −1.3058
Normalized ES: −1.4228
Normalized ES: −1.5062
Normalized ES: −1.3573
Positively correlated
Negatively correlated
O-MET
O-NR
O-CTRL
F
Rescue DEGs ∩ Aging Atlas

Figure S3

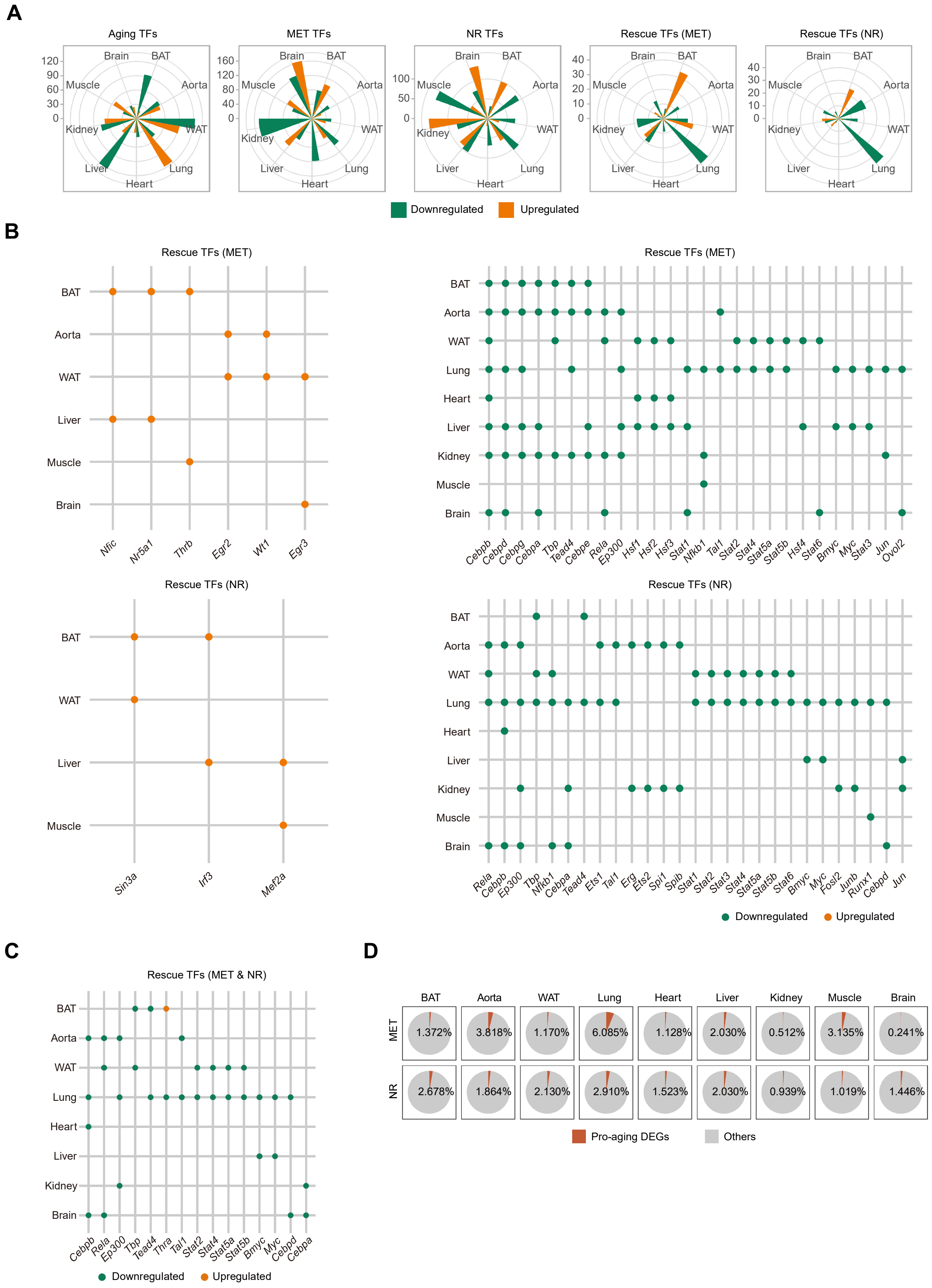
A
Aging TFs
MET TFs
NR TFs
Rescue TFs (MET)
Rescue TFs (NR)
Brain
BAT
Aorta
WAT
Lung
Heart
Liver
Kidney
Muscle
Downregulated
Upregulated
B
Rescue TFs (MET)
Nfic
Nr5a1
Thrb
Egr2
Wt1
Egr3
Cebpb
Cebpd
Cebpg
Cebpa
Tbp
Tead4
Cebpe
Rela
Ep300
Hsf1
Hsf2
Hsf3
Stat1
Nfkb1
Tal1
Stat2
Stat4
Stat5a
Stat5b
Hsf4
Stat6
Bmyc
Myc
Stat3
Jun
Ovol2
Rescue TFs (NR)
Sin3a
Irf3
Mef2a
Rela
Cebpb
Ep300
Tbp
Nfkb1
Cebpa
Tead4
Ets1
Tal1
Erg
Ets2
Spi1
Spib
Stat1
Stat2
Stat3
Stat4
Stat5a
Stat5b
Stat6
Bmyc
Myc
Fosl2
Junb
Runx1
Cebpd
Jun
C
Rescue TFs (MET & NR)
Cebpb
Rela
Ep300
Tbp
Tead4
Thra
Tal1
Stat2
Stat4
Stat5a
Stat5b
Bmyc
Myc
Cebpd
Cebpa
D
MET
NR
1.372%
3.818%
1.170%
6.085%
1.128%
2.030%
0.512%
3.135%
0.241%
2.678%
1.864%
2.130%
2.910%
1.523%
2.030%
0.939%
1.019%
1.446%
Pro-aging DEGs
Others

Figure S4

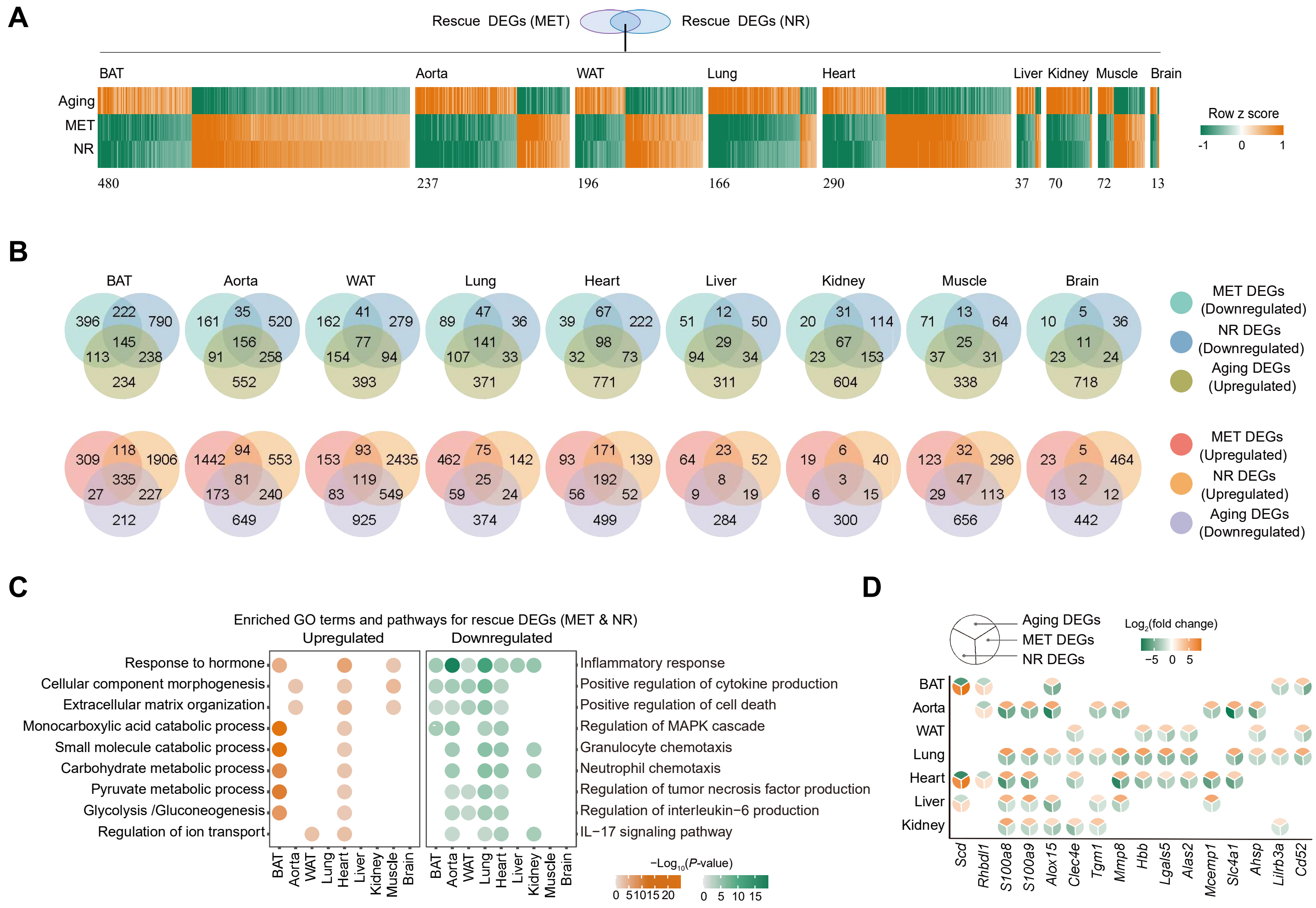
A
Rescue DEGs (MET)
Rescue DEGs (NR)
BAT
Aorta
WAT
Lung
Heart
Liver
Kidney
Muscle
Brain
Aging
MET
NR
Row z score
-1
0
1
480
237
196
166
290
37
70
72
13
B
MET DEGs (Downregulated)
NR DEGs (Downregulated)
Aging DEGs (Upregulated)
MET DEGs (Upregulated)
NR DEGs (Upregulated)
Aging DEGs (Downregulated)
C
Enriched GO terms and pathways for rescue DEGs (MET & NR)
Upregulated
Downregulated
Response to hormone
Cellular component morphogenesis
Extracellular matrix organization
Monocarboxylic acid catabolic process
Small molecule catabolic process
Carbohydrate metabolic process
Pyruvate metabolic process
Glycolysis /Gluconeogenesis
Regulation of ion transport
Inflammatory response
Positive regulation of cytokine production
Positive regulation of cell death
Regulation of MAPK cascade
Granulocyte chemotaxis
Neutrophil chemotaxis
Regulation of tumor necrosis factor production
Regulation of interleukin−6 production
IL−17 signaling pathway
−Log10(P-value)
D
Aging DEGs
MET DEGs
NR DEGs
Log2(fold change)
Scd
Rhbdl1
S100a8
S100a9
Alox15
Clec4e
Tgm1
Mmp8
Hbb
Lgals5
Alas2
Mcemp1
Slc4a1
Ahsp
Lilrb3a
Cd52
